# Next-Generation Sequencing Revealed Disease-Causing Variants in Two Genes in a Patient With Combined Features of Spherocytosis and Antley-Bixler Syndrome With Genital Anomalies and Disordered Steroidogenesis

**DOI:** 10.3389/fgene.2020.00976

**Published:** 2020-08-21

**Authors:** Fuying Song, Shunqiao Feng, Xiang Shen, Mu Du, Hui Yin, Rong Liu, Xiaobo Chen

**Affiliations:** ^1^Department of Endocrinology, Capital Institute of Pediatrics, Beijing, China; ^2^Department of Hematology, Capital Institute of Pediatrics, Beijing, China; ^3^Running Gene Inc., Beijing, China

**Keywords:** combined disease symptoms, multi-system involved, congenital adrenal hyperplasia, hereditary spherocytosis, POR, ANK1

## Abstract

Conventionally, patients with combined rare diseases are often difficult to diagnose. This is because some clinicians tend to consider the multiple disease symptoms as the presentation of a complicated “syndrome.” This pattern of thinking also confines their way of filtering pathogenic mutations. Some real pathogenic mutations might be ignored due to not covering all disease presentations. Here we report the case of a girl who was suffering from spherocytosis and Antley-Bixler syndrome with genital anomalies and disordered steroidogenesis. She remained undiagnosed even after targeted gene detection before. However, after performing next-generation sequencing and analyzing the sequencing data, we identified two mutations: c.2978T > A in *ANK1* and c.1370G > A in *POR*. Our findings and experiences in diagnosing these mutations could contribute to the existing knowledge on the clinical and genetic diagnosis of patients with disease presentations in multiple systems.

## Introduction

Hereditary spherocytosis (OMIM 182900), with a prevalence of 1/2,000, is a hemolytic disorder characterized by the presence of spherical erythrocytes on a peripheral blood smear (Gallagher, 2005; Perrotta et al., 2008; Yocum et al., 2012). Clinical presentations include anemia, jaundice, and splenomegaly with heterogeneous severity (Perrotta et al., 2008). Genes related to hereditary spherocytosis include *ANK1*, *SLC4A1*, *SPTB*, *EPB42*, and *SPTA*. Among them, *ANK1* is the most common disease-causing gene (Yocum et al., 2012). The *ANK1* gene (OMIM^∗^612641) encodes human erythroid ankyrin containing 1,880 amino acids. It combines tetramers of spectrin with the cytoplasmic domain of band three to form a spectrin-actin based membrane skeleton. This skeleton further interacts with the plasma membrane to stabilize the erythrocyte membrane (Gallagher, 2005). Defects in ankyrin synthesis or structure might result in instability of the erythrocyte membrane, thus causing hereditary spherocytosis.

Congenital adrenal hyperplasia (CAH) is a group of autosomal recessive disorders characterized by impaired cortisol biosynthesis due to deficiencies in the various enzymes in the adrenal steroidogenesis pathway (Hannah-Shmouni et al., 2017; El-Maouche et al., 2017; Witchel, 2017). The candidate genes include *CYP21A2*, *CYP11A1*, *CYP11B1*, *CYP17A1*, *HSD3B2*, *StAR*, and *POR* (El-Maouche et al., 2017; Hannah-Shmouni et al., 2017). CAH due to *POR* deficiency is a rare variant of CAH. *POR* (OMIM^∗^124015) encodes cytochrome P450 oxidoreductase. It is a flavoprotein that donates electrons to all microsomal P450 enzymes, including 17OH and 21OH. Therefore, combined 17OH and 21OH deficiency could be observed in the patients (El-Maouche et al., 2017). The female patients with *POR* deficiency always present prenatal virilization, as the alternative pathway of producing androgens is not blocked (Arlt et al., 2004). Besides, some of these patients present skeleton malformations, such as craniosynostosis, radioulnar, and midface hypoplasia. This skeleton malformation presentation involved a form of CAH called Antley-Bixler syndrome or Antley-Bixler syndrome with genital anomalies and disordered steroidogenesis (OMIM 201750) in OMIM.

Herein, we report a Chinese patient presenting combined features of CAH and Antley-Bixler syndrome. Deficiencies in two related genes – *ANK1* and *POR* – were identified by whole-exome sequencing. To our knowledge, patients carrying multiple causative gene mutations are uncommon since most patients are diagnosed as having a “syndrome” when they have symptoms involving multiple systems. Here, we share our experience in the diagnosis of this rare case.

## Case Presentation

### Subject and Clinical Features

The study was approved by the ethics committee of the Capital Institute of Pediatrics. Written informed consent was obtained from the patient’s parents for the publication of this report and any accompanying images.

The patient was admitted to our hospital due to CAH. The patient was a female aged 10 years and six months. She was the first child born to non-consanguineous parents. She was born by normal cesarean delivery with a birth weight of 3,200 g (P25–P50) and a birth height of 51 cm (P50–P75). Her mother was diagnosed with polycystic ovary syndrome during pregnancy. Our patient presented clitoromegaly at birth with chromatosis of the vulva but not the skin. An ovarian cyst with a size of 5.7 × 3.7 × 4.3 mm was observed on the patient’s right ovary when performing a pelvic ultrasound 23 days after birth. A karyotype test showed a normal karyotype of 46 autosomal chromosomes and two sex chromosomes. A biomedical test indicated slight abnormality with increased K+ (5.7 mmol/l) and decreased Na+ (133.9 mmol/l). The concentration of the adrenocorticotropic hormone (ACTH) was 3.09 pg/ml and cortisol was 4.4 μg/dl at 8 am. In a sex hormone test, the luteinizing hormone (LH) was 38.9 μl U/ml, the follicle-stimulating hormone (FSH) was 7.7 l U/l, testosterone (T) was 123.9 ng/dl, and estradiol (E2) was 36.5 pg/dl. When the patient was three years old, she had a clitoridectomy. At the age of five, the patient received gene detection by a gene panel in another hospital. However, the gene result revealed no possible pathogenic mutations (the original samples and results are missing). An ACTH stimulation test identified cortisol (10.5 μg/dl), Testosterone (16.4 ng/dl), and 17-OHP (19.050 ng/ml). Hydrocortisone (5 mg/bid) has been used to treat the patient until present, although no significant progress has been observed. When the patient was 5 years and 6 months old, she was admitted to our hospital due to “fever, fatigue, and pale skin lasting more than 1 week.” She was found to have hepatosplenomegaly during a physical test, her liver was palpable at 2 cm and her spleen was palpable at 4 cm below the costal margin. In a routine blood test, her white blood cell count (WBC) was 13.67 × 10 E9/l, hemoglobin (Hb) was 49 g/l, and reticulocyte (Ret) was 0.065. A blood smear revealed that the patient’s proportion of spherocytes was 7% and seven nucleated red blood cells per 100 white blood cells could be counted. In an erythrocyte osmotic fragility test, hemolysis began at 0.56% salt solution (normal control at 0.42%) and complete hemolysis began at 0.32% salt solution (normal control at 0.22%). In liver function tests, total bilirubin (TB) was 30.9 μmol/l, direct bilirubin (DB) 12.1 μmol/l, and lactate dehydrogenase (LDH) 382 U/l. A routine bone marrow test showed significantly active bone marrow proliferation and active erythroid proliferation, as well as normal folic acid, vitamin B12, ferritin, glucose phosphate isomerase, glucose-6-phosphate dehydrogenase, and pyruvate kinase tests. Moreover, Coomb’s test showed negative results. The patient was then clinically diagnosed with hereditary spherocytosis. The patient was then not treated and her jaundice relapsed. She received an ultrasound-guided percutaneous cholecystolithotomy and partial splenic artery ligation when she was 10 years old. The patient’s condition soon improved and her jaundice did not return.

#### Physical Examination During Admission

**FIGURE 1:**
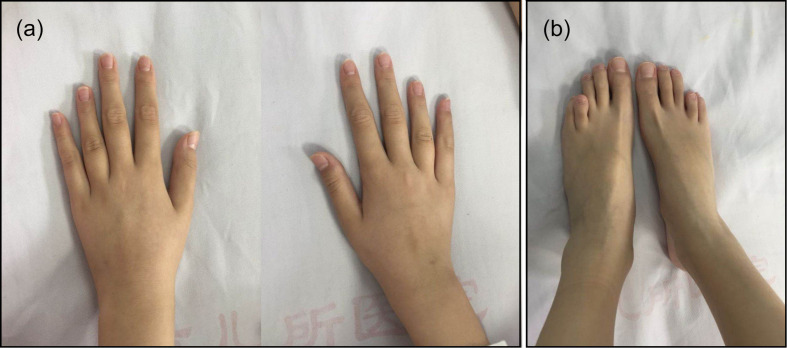
Arachnodactyly with long and slender fingers **(a)** and toes **(b)** could be observed. The bilateral fourth and fifth metacarpal bones and metatarsal bones were all short.

The patient’s weight was 29 kg (P25–P50) and her height was 144.5 cm (P25–P50). Her blood pressure was 100/60 mmHg and her bone age was normal, as was her mental status. A café-au-lait spot with a diameter of 2 cm was observed at the patient’s waist. She was in Tanner stage I of puberty. Arachnodactyly with long and slender fingers (Figure 1a) and toes (Figure 1b) was observed. The fourth and fifth metacarpal bones of the patient’s hands were short and thick. Stiffness was observed in the elbow and knee joints. The patient could not make a fist due to joint contracture. Bony swelling was observed on the patient’s sacrum.

#### Imaging Tests

X-ray examination: foot AP axial view: the bilateral fourth and fifth metatarsal bones of the patient were short, the fourth phalanxes were short, and the bone mineral density of both feet was relatively low. Knee AP view: spiking of the bilateral tibial intercondylar eminence was observed. Lumbar spine AP view: slight scoliosis. Elbow AP view: mean trabecular plate density was relatively low. Contrast-enhanced CT scanning: no obvious abnormality was observed in the adrenal glands. However, the intrahepatic bile duct expansion, common bile duct expansion, and splenomegaly were observed.

#### Laboratory Analyses

Auxiliary examinations: the patient had normal serum electrolytes, while a sex hormone test showed that her FSH was 3.23 IU/l, her LH was less than 0.1 IU/l, and her E2 was less than 18.35 pmol/l. She had testosterone (T) of 559 ng/ml (14–76), prolactin (PRL) of 28.61 ng/ml, progesterone (PROG) of 0.25 nmol/l, and parathyroid hormone (PTH) of 14.5 pg/ml. Her 17-hydroxyprogesterone (17-OHP) was 2.14 ng/ml, her dehydroisoandrosterone (DHEA) was 0.8 ng/ml, her androstenedione was less than 0.3 ng/ml, and her dihydrotestosterone was 13.86 pg/ml. Her cortisol at 8 am, 4 pm, and 0 am were 2.25, 4.9, and 15.71 μg/dl, respectively. Her renin, angiotensin, 1-angiotensin, and 2-aldosterone were 3.08 ng/ml, 37 pg/ml, 40 pg/ml, and 132 pg/ml, respectively at recumbent position, and 3.69 ng/ml, 23 pg/ml, 37 pg/ml, and 282 pg/ml, respectively at erect position. Uterus and ovaries ultrasound images demonstrated that the length of the patient’s uterus was 1.65 cm with a diameter of 0.5 cm, and her cervix length was 1.6 cm. The patient’s endometrium could not be delineated clearly, not excluding the possibility that a primordial uterus existed.

#### Treatment

An ultrasound-guided percutaneous cholecystolithotomy and partial splenic artery ligation were performed. After the operation, the intrahepatic bile duct and common bile duct were expanded, the distal common bile duct narrowed, diffuse enlargement was observed, and the middle and lower parts of the spleen were infracted. Hydrocortisone (5 mg/bid) was taken orally by the patient for 6 days until she was discharged.

## Methods

### Whole-Exome Sequencing

Proband’s DNA was sequenced to discover the causal gene. Next-generation sequencing was performed by Running Gene Inc. following the manufacturer’s protocol. DNA was isolated from peripheral blood using a DNA Isolation Kit (Bioteke, AU1802) and 1 μg genomic DNA was fragmented into 200–300 bp lengths using a Covaris Acoustic System. The DNA fragments were then processed by end-repairing, A-tailing and adaptor ligation, a four-cycle pre-capture polymerase chain reaction (PCR) amplification, and targeted sequences capture. Captured DNA fragments were eluted and amplified by 15-cycle post-capture PCR. The final products were sequenced with 150 bp paired-end reads on an Illumina NovaSeq platform according to the standard manual.

The raw data converted by NovaSeq were filtered and aligned against the human reference genome (hg19) using the BWA Aligner^1^. The single-nucleotide polymorphisms (SNPs) were called by using the Genome Analysis ToolKit (GATK) software^2^. Variants were annotated using ANNOVAR^3^. The effects of single-nucleotide variants (SNVs) were predicted by the SIFT, Polyphen-2, and MutationTaster programs. All variants were interpreted according to the standards for the interpretation of sequence variations recommended by ACMG (American College of Medical Genetics and Genomics) and categorized as pathogenic, likely pathogenic, variants of unknown clinical significance (VUS), likely benign, or benign. The associated phenotypic features of candidate genes were analyzed against the patient’s phenotype. Core phenotypes were extracted and used to acquire a gene list of the virtual panel by the OMIM database^4^ and Mingjian (211.149.234.157/login). Re-annotation was conducted according to the virtual panel.

### Sanger Sequencing

The candidate causal genes discovered via whole-exome sequencing (WES) were confirmed by Sanger sequencing, and co-segregation analyses among the family were also conducted. The primers were designed using Primer Premier 5.0 (Premier Biosoft). PCR was conducted to amplify the fragments covering the mutated sites. The PCR products were further purified with a Zymoclean PCR Purification Kit and then sequenced with an ABI 3730 DNA Sequencer. The Sanger sequencing results were analyzed by Chromas Lite v2.01 (Technelysium Pty Ltd., Tewantin, QLD, Australia).

## Results

**FIGURE 2:**
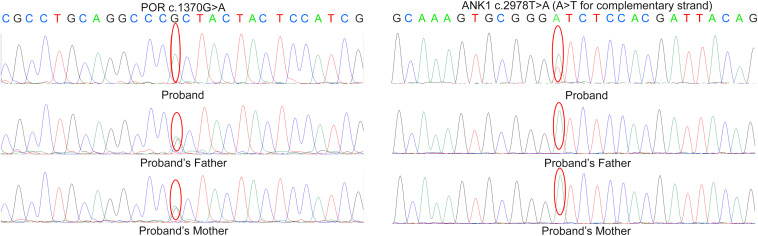
Sanger sequencing result in the proband’s family. According to the result, the parents of proband are both the carrier of c.1370G > A variant in *POR* but doesn’t carry the mutation c.2978T > A in *ANK*.

A heterozygous mutation (c.2978T > A) in *ANK1* (NM_000037) and a homozygous mutation (c.1370G > A) in *POR* (NM_000941) were identified by next-generation sequencing (Supplementary Figure S1), which resulted in amino acid alterations p.I993N and p.R457H, respectively. According to validation by Sanger sequencing in the proband’s family, the proband’s parents were both carriers of heterozygous mutation c.1370G > A in the *POR* gene. Neither parent had mutation c.2978T > A in *ANK1* (Figure 2).

According to the results, variant c.2978T > A in *ANK1* was proven to be a *de novo* mutation by Sanger sequencing (PS2). This variant was also absent in the control (PM2) and is predicted to be deleterious by multiple *in silico* algorithms (PP2): it was predicted to be disease-causing by MutationTaster (>0.99), deleterious by PROVEAN (−6.85), damaging by SIFT (0.00), and probably damaging by Polyphen-2 (1.000). The patient’s phenotype was also specific for the disease, as she presented spherocytosis (PP4). Therefore, according to ACMG standards, this variant could be interpreted as likely pathogenic. Mutation c.1370G > A is a previously established pathogenic variant (PS1), and there is well-established functional studies supported the damaging effect of this variant (PS3). For instance, this mutation is classified as pathogenic according to ACMG guidelines.

## Discussion and Conclusion

Hereditary spherocytosis is a common congenital hematologic disorder and also a common reason for inherited chronic hemolysis in Western countries. Hereditary spherocytosis has heterogeneous clinical presentations. In most cases, patients are asymptomatic or only present anemia. However, for patients have mild disease courses, 20–30% of these patients have mild splenomegaly and reticulocytosis, while for patients have moderate disease courses, 60–70% of them typically presented asymptomatic anemia, splenomegaly, and jaundice during infections in their childhood (Perrotta et al., 2008).

The structure of the erythrocyte membrane contains band-3 protein; protein 4.1; protein 4.2; ankyrin; spectrin; glycophorin A, B, C, and D; Rhesus (Rh) complex; CD47; Landsteiner-Wiener glycoprotein; dematin; tropomyosin; adducin; and tropomodulin. Deficiency in any of these proteins may result in the disassociation of the erythrocyte membrane structure and subsequently reduce the surface-to-volume ratio, which finally causes hereditary spherocytosis (Eber and Lux, 2004). Since ankyrin-1 encoded by ANK1 mediates the linkage of band-3 protein with spectrin, it plays an essential role in the stabilization of the membrane. Deficiency in ankyrin-1 protein may also result in the decreased assembly of spectrin, regardless of the spectrin synthesis (Hanspal et al., 1991).

**FIGURE 3:**
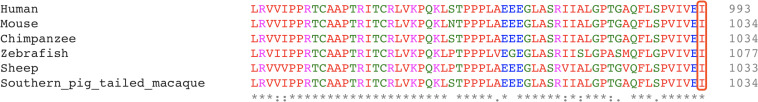
Sequencing alignment of residue I993 in ANK1 protein. Result shows that this residue is extremely conserved among species.

In our case, the patient presented hereditary spherocytosis due to mutation in *ANK1*. The proband was identified with heterozygous mutation c.2978T > A, which results in amino acid change p.I993N. Residue I993 is a highly conservative residue in ankyrin-1 protein among several species (Figure 3). The conservation of this residue indicates its importance in evolution. Besides, isoleucine is a non-polar neutral amino acid, while asparagine is a polar neutral amino acid. The alteration in polarity might change the interaction between amino acids within the structure or between ankyrin and other proteins, thus affecting the function of ankyrin. Moreover, residue resides in a domain called ZU5 (from residue 911 to residue 1,015). Experiment of Mohler et al. (2004) shows that this domain could help in the binding of spectrin. The occurrence of variation in this domain may disrupt the association between β-spectrin and ankyrin-1, thus dissociating the erythrocyte membrane structure.

Congenital adrenal hyperplasia refers to a series of disorders in adrenal steroidogenesis that can affect the biogenesis of glucocorticoid, mineralocorticoid, or sex steroid production. CAH consists of two forms: a severe form and a mild form. The severe form affects nearly one in 15,000 births worldwide, while the prevalence of the mild form is one in 1,000 or even higher in certain ethnic groups (Merke and Kabbani, 2001).

**FIGURE 4:**
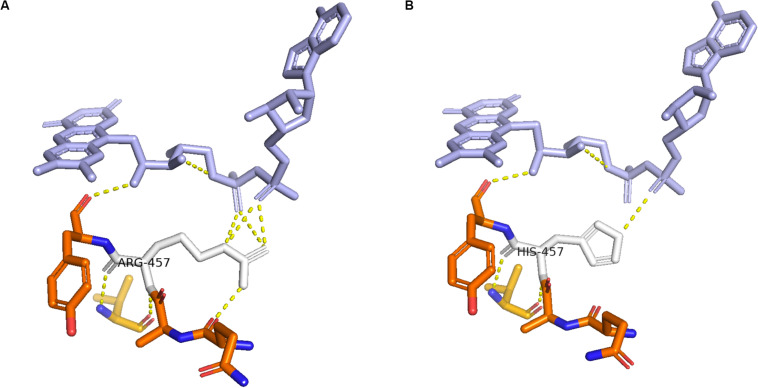
Structure of cytochrome P450 oxidoreductase before and after mutation. **(A)** Crystal structure of wild-type P450 oxidoreductase (pbd id:5FA6). **(B)** Crystal structure of mutant P450 oxidoreductase (pdb id:3QFR). The attraction between P450 oxidoreductase and FAD was found significantly decreased.

Enzymes involved in cortisol biosynthesis include 21-hydroxylase, 11β-hydroxylase, 17α-hydroxylase, 3β-hydroxysteroid dehydrogenase (type II), steroidogenic acute regulatory protein, P450 cholesterol side-chain cleavage enzyme, and P450 oxidoreductase (*POR*). The initial two enzymes in cortisol biosynthesis, 17OH and 21OH, are functionally active only when the electron transport cytochrome P450 oxidoreductase exists. Therefore, patients with *POR* deficiency present combined 17OH and 21OH deficiency. The patient in our study was identified as carrying homozygous mutation c.1370G > A, which caused amino acid variation p.R457H. This mutation is a reported variant that is located in the FAD binding domain. The guanidinium group of residue R457 was proven to form a salt bridge with the pyrophosphate group of FAD (Wang et al., 1997; Xia et al., 2011). Alteration in H457 weakens the interaction between FAD and POR according to the molecule structure model (Figure 4). Since this variant is a known disease-causing mutation, the clinical presentation is also summarized as Table 1 (Adachi et al., 2004; Arlt et al., 2004; Flück et al., 2004; Fukami et al., 2005; Bai et al., 2017). According to the summarized information, nearly all patients carrying mutation R457H are diagnosed as having Antley-Bixler syndrome, except for one patient, who did not present any skeletal malformation. Our patient presented arachnodactyly, multiple digital contractures, scoliosis, and clitoromegaly, as in most of the reported patients; therefore, she could be diagnosed as having Antley-Bixler syndrome.

**TABLE 1:** Summarized clinical information of patients carrying mutation c.1370G > A (p.R457H) in the POR gene.

Report	Sex		Clinical Presentation
Adachi et al. (2004)	M	Com Het	Cardiac arrest due to upper airway obstruction twice during infancy. Craniosynostosis, radiohumeral synostosis, carpal and tarsal bone fusion, malformed and simple ear, arachnodactyly.
Adachi et al. (2004)	F	Com Het	Bilateral radiohumeral synostosis, midfacial hypoplasia, malformed ears, and genital ambiguity including clitoromegaly and labial fusion. No secondary sex characteristics appeared at 11 years of age.
Fukami et al. (2005)	M	Com Het	Craniosynostosis, midfacial hypoplasia, multiple digital joint contractures, arachnodactyly, scoliosis at 13 years of age, micropenis, cryptorchidism, hypospadias.
Fukami et al. (2005)	M	Com Het	Craniosynostosis, midfacial hypoplasia, radiohumeral synostosis at 10 years of age, multiple digital joint contractures, arachnodactyly, micropenis, cryptorchidism.
Fukami et al. (2005)	M	Com Het	Craniosynostosis, midfacial hypoplasia, multiple digital joint contractures, arachnodactyly, cryptorchidism.
Fukami et al. (2005)	F	Hom	Craniosynostosis, midfacial hypoplasia, multiple digital joint contractures, clitoromegaly, labial fusion.
Fukami et al. (2005)	F	Het	Craniosynostosis, midfacial hypoplasia, multiple digital joint contractures, arachnodactyly, labial fusion.
Fukami et al. (2005)	F	Hom	Craniosynostosis, midfacial hypoplasia, multiple digital joint contractures, clitoromegaly, labial fusion.
Fukami et al. (2005)	F	Com Het	Craniosynostosis, midfacial hypoplasia, conduction deafness at 13 years of age, multiple digital joint contractures, arachnodactyly, clitoromegaly, labial fusion.
Fukami et al. (2005)	F	Hom	Proximal interphalangeal joint, clitoromegaly.
Arlt et al. (2004)	F	Com Het	Clubfeet, ambiguous genitalia, clitoromegaly, labial fusion, marfanoid habitus, scoliosis, arachnodactyly, dysplastic ears, slim limbs, unilateral oophorectomy.
Flück et al. (2004)	M	Com Het	Craniosynostosis, hypertelorism, mild choanal narrowing, radiohumeral synostosis, midface hypoplasia, arachnodactyly, rocker-bottom feet, clitoromegaly, labial fusion.
Bai et al. (2017)	F	Hom	Labial fusion, ovarian cyst without special skeletal deformities.

*Com Het, compound heterozygous; Hom, homozygous; Het, heterozygous.*

In this study, we reported a girl presenting both spherocytosis and Antley-Bixler syndrome with genital anomalies and disordered steroidogenesis. Patients presenting more than one diseases, especially hereditary diseases, are not a common affair for the clinicians. Therefore, when handling cases presenting multi-system symptoms, some of the clinicians would tend to treat it as a rare syndrome not several separated diseases. This thought might impede the analysis of genetic tests. We hope our experience in diagnosing this case would delight other clinicians in handling patients with disease presentations in multiple systems. Moreover, the cooperation between doctors in different department is also important. In our case, the clitoromegaly of the patient was treated when she was 3 years old in other hospital without a clear diagnosis. She was admitted to the hematological department of our hospital at the age of 5 only due to the complaint of spherocytosis. However, since the patient was found to have arachnodactyly during admission she was then sent to the department of endocrinology. After the next-generation sequencing and the examination of clinicians in both departments, she was finally diagnosed.

## Data Availability Statement

The raw data supporting the conclusions of this article will be made available by the authors, without undue reservation.

## Ethics Statement

The study was approved by the Ethics Committee of the Capital Institute of Pediatrics. Written informed consent was obtained from the patient’s parents for the publication of this report and any accompanying images.

## Author Contributions

SF and RL were in charge of the diagnosis of spherocytosis and also the writing works concerning the presentations of spherocytosis. They also gave advices when doing the gene analysis. FS, MD, HY, and XC were responsible for the diagnosis of CAH and all the description parts of CAH in this article. They also gave advices when doing the gene analysis. XS took the charge of performing gene sequencing and the analysis and also the genetic parts of this article. All authors agreed to be accountable for the content of the work.

## Conflict of Interest

XS was employed by the company Running Gene Inc. The remaining authors declare that the research was conducted in the absence of any commercial or financial relationships that could be construed as a potential conflict of interest.

## Footnotes

1 http://bio-bwa.sourceforge.net/

2 https://software.broadinstitute.org/gatk/

3 http://annovar.openbioinformatics.org/en/latest/

4 http://omim.org/
