# Supplementary material for: Next-Generation Sequencing Revealed Disease-Causing Variants in Two Genes in a Patient With Combined Features of Spherocytosis and Antley-Bixler Syndrome With Genital Anomalies and Disordered Steroidogenesis
Source: Front Genet. 2020 Aug 21;11:976. doi: 10.3389/fgene.2020.00976 (PMC7472872; doi:10.3389/fgene.2020.00976)
Supplement: Supplementary file 1 [file Image_1.pdf]

Supplementary Material

1.1 Supplementary Figures

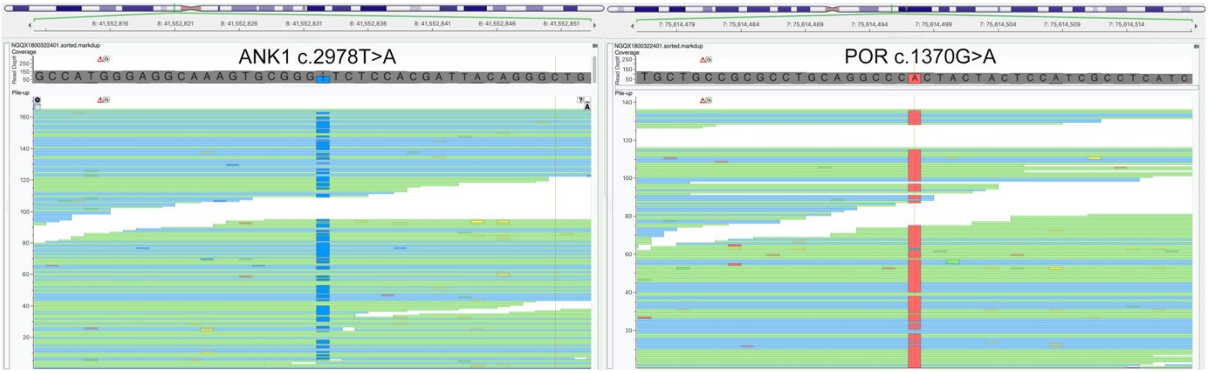

Figure S1. Next-Generation Sequencing result of the proband. According to the result, this patient carries heterozygous mutation c.2978T>A in *ANK1* gene and homozygous mutation c.1370G>A in *POR* gene.s
